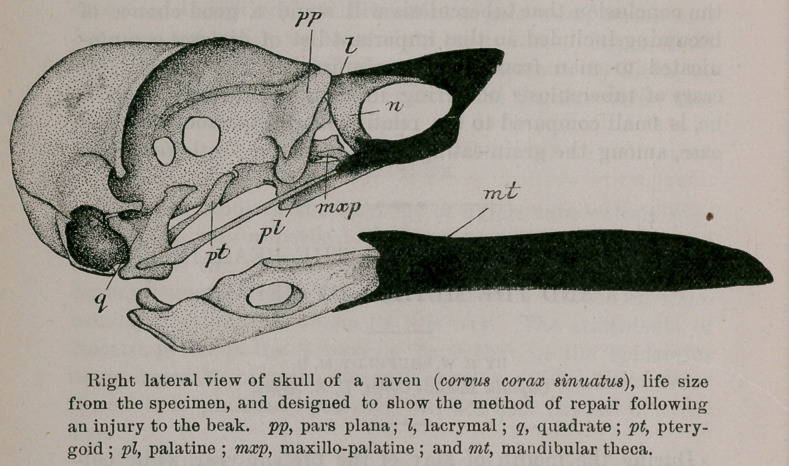# On Injuries of the Beak in Birds, and the Method of Repair

**Published:** 1886-10

**Authors:** R. W. Shufeldt

**Affiliations:** Captain Medical Corps, U. S. Army


### Art. XVII.—ON INJURIES OF THE BEAK IN BIRDS, AND THE METHOD OF REPAIR.

BY R. W. SHUFELDT, M. D.
*Captain Medical Corps, U. S. Army.*

During the month of May of the present year, while collecting about Fort Wingate, New Mexico, I often noticed among the troops of ravens (*corvus corax sinuatus*), that were to be seen at any time during the day about the gardens and slaughter house of the station, one that had evidently had his superior mandible shot away. This no doubt had been done with a rifle ball by one of the men, as they constantly fired at them with their rifles for practice. Nearly a month after I first noticed this bird, I was in need of a raven for my anatomical studies of the group, and a good opportunity offering one morning, I fired upon a number of them, killing one with each barrel, and, curious enough, upon picking up my specimens, I at once discovered that I had bagged the deformed one in question.

This bird had indeed met with a serious injury, but the natural repair was so complete and so interesting, that I prepared the skull of the individual and made the following record of the case.

I found the bird to be well nourished, though not fat, and with a beautiful suit of plumage, black and glossy.

The ball had carried away the upper bill just forward of the nostrils. This had had a notable effect upon the form of the lower mandible, for the anterior third of this part of the

Right lateral view of skull of a raven (*corvus corax sinuatus*), life size from the specimen, and designed to show the method of repair following an injury to the beak. *pp*, pars plana; *l*, lacrymal; *q*, quadrate; *pt*, pterygoid; *pl*, palatine; *mxp*, maxillo-palatine; and *mt*, mandibular theca.

skull was evidently much compressed laterally, and the superior margins of the horny theca covering it, curled over towards each other and the median line. The subcylindrical channel thus formed was tightly packed with earth, no doubt rammed in there by the efforts of the bird to pick things up from the ground. I had to soak this mandible some time in water before this hard plug came away, and it is fair to presume that it caused this raven no special inconvenience, and it never made any attempt to clear it.

If we examine a normal skull of a raven, we find that the superior mandible immediately in front of the osseous nasal apertures is hollow and quite densely filled in with cancellous tissue. The side of the mandible here has an altitude of about 1 centimetre and 2 or 3 millimetres, while its base, in the roof of the mouth, measures a few millimetres more than this. Here is where the injury took place in our specimen. When the bullet cut the bill away, the anterior aspect, it will thus be seen, was of a triangular outline, and of an area agreeing with the approximate measurements we have given for the base and altitude of the parts in question.

The outer edge of this triangle, of course, was the free edge of the horny, integumental theca that overlaid the superior smandible. Now this open wound, with its firm bony walls would certainly have been a source of constant danger to this bird, had not the method nature adopted to seal it been of a very effective kind.

As it healed however, the cut edges of the osseous bill met each other in the median line, and here very perfectly, for their entire vertical distance, united completely by bony union. The stump thus formed deflected slightly to the left, while the cut anterior extremities of the palatines in the roof of the mouth, curled slightly towards each other.

When we see that the bone was thus able to so prettily close in such an ugly injury, it is not at all surprising that the horny covering followed suit. And this is just what took place, for the cut margins of this latter tissue growing to meet the occasion, it succeeded in entirely encasing the stump formed by the bone.

The result of nature's surgery in this case, then, has been a repair that practically leaves the injured part in such a condition, that it did not submit the individual to the danger of subsequent inflammations, while the form of the resulting stump is as useful a one as could possibly be expected to have followed from a wound of such a character.

Injuries of this kind in birds must be quite rare, for in a collecting experience of twenty years or more, I cannot recall a similar instance, although it is by no means an uncommon thing to find that they have sustained injuries in all other parts of the body, where too, as is well known, we find other interesting examples of the methods of repair.

## Figures and Tables

**Figure f1:**